# Human Steering Control Under Unpredictable Disturbances

**DOI:** 10.1111/ejn.70605

**Published:** 2026-06-25

**Authors:** Jo‐Yu Liu, James R. H. Cooke, Luc P. J. Selen, W. Pieter Medendorp

**Affiliations:** ^1^ Donders Institute for Brain, Cognition and Behaviour Radboud University Nijmegen the Netherlands

**Keywords:** disturbance, optimality, robustness, steering, vestibular

## Abstract

There are multiple methods for controlling dynamical systems under external disturbances, with some emphasizing robust performance and others prioritizing efficiency. Here, we characterized the control strategies of human steering behavior under unpredictable disturbances. Using a self‐steerable motion platform, participants (*n* = 22) steered a vehicle along roads of different widths (narrow, medium, and wide) while experiencing random, time‐varying physical perturbations. Steering control strategies were assessed in terms of the frequency‐dependent gain and phase of compensatory steering for the different road widths. Participants' road‐keeping performance depended on the road width. When transitioning from narrow to wide roads, participants carried over their existing control strategy, suggesting a control policy that prioritizes robustness. This persistence was reflected in the vehicle spending more time within the road boundaries of the wide road than on the narrow ones. When moving from wide to narrow roads, participants again largely maintained their control strategy, albeit with some modest modulation by road width. In the frequency domain, road order did not affect the gain but did modulate the phase of control, suggesting that any control policy adjustments occurred primarily in the timing between perturbation and response. Together, these findings suggest that feedback gains for steering under random perturbations reflect a neural control strategy mainly tuned for robustness, with only a modest influence of efficiency.

## Graphical abstract

Humans steered a vehicle along roads of varying width while experiencing random, time‐varying physical perturbations. Frequency analysis showed that road width did not affect gain but did modulate the phase of control, suggesting that any control policy adjustments occurred primarily in the timing between perturbation and response. These findings suggest that human steering control under random disturbances is predominantly tuned for robustness rather than efficiency.

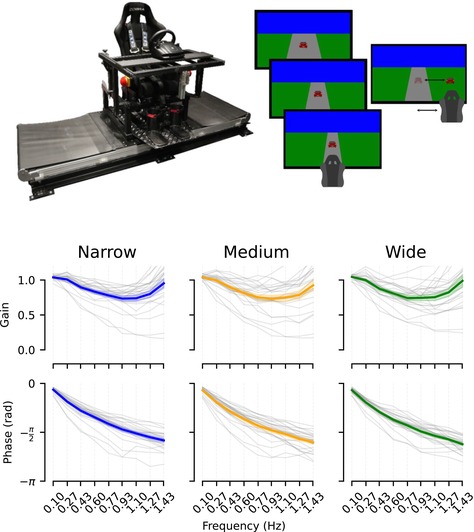

**Abbreviations**
ANOVA: analysis of variance
LQG: linear‐quadratic‐gaussian
OFC: optimal feedback control
RMSE: root‐mean‐square error
SE: standard error

## 1 Introduction

Effective motor control is essential for safe and precise navigation, particularly in dynamic settings such as driving. Successfully steering a vehicle requires continuous monitoring and adjustment to remain within road boundaries, avoid obstacles, and respond to unpredictable changes in the environment (Land and Horwood 1995; Salvucci and Gray 2004; Markkula et al. 2023). Proficiency in steering control depends on the sensorimotor system's ability to cope with sensory and motor noise (Faisal et al. 2008), neural transmission delays (Oostwoud Wijdenes and Medendorp 2017), the inherent dynamics of sensorimotor transformations (Todorov 2004; Franklin and Wolpert 2011), and any external disturbances of the system or environment (Maurus et al. 2023).

An influential theoretical framework that provides a computational solution to many of these difficulties is optimal feedback control (OFC) (Todorov and Jordan 2002; Scott 2012). The core idea is that the brain combines sensory information and predictions from internal models to compute an optimal estimate of the state of the system. This state estimate is then used to compute a motor command involving feedback gains. These gains are optimized to minimize an expected cost function, which can encompass energy, accuracy, time, and related factors. Because these components are difficult to disentangle behaviorally, they are subsumed under the single notion of cost. The optimization process yields a cost‐efficient controller whose feedback gains are tuned to task demands: higher when precise control is required and lower when control is less critical.

Recently, OFC has been successfully applied to models of human steering, demonstrating its ability to use internal models to integrate visual, vestibular, and neuromuscular dynamics while accounting for noise and delays to generate optimal control actions (Nash and Cole 2020; Liu et al. 2025). However, OFC assumes that system dynamics and disturbance statistics are perfectly known. Consequently, unmodeled perturbations, i.e., those outside the system representation, cannot be directly accommodated.

As an alternative, robust control theory (Başar and Bernhard 1995; Ueyama 2014; Bian et al. 2020) focuses on maintaining a stable system despite model uncertainties and external disturbances. It emphasizes system stability (robustness) over efficiency (cost savings). Robust control has recently been applied to model human motor behavior under unpredictable disturbances (Ueyama 2014; Crevecoeur et al. 2019; Bian et al. 2020; Maurus et al. 2023). Experimentally, Crevecoeur et al. (2019) showed that an unpredictable disturbance during a single reach increases movement speed and feedback gains in subsequent trials, independent of perturbation direction. Similar observations were made by Maurus et al. (2023), who forced participants into a robust control strategy to handle continuous, unpredictable, and time‐varying perturbations during a reach. Thus, the nervous system seems to shift to a more robust control policy to cope with unmodeled disturbances, either because they have never been encountered or are unpredictable.

It is important to point out that OFC and robust control are two extremes on a continuum. The former assumes only uncertainty in terms of unbiased Gaussian noise, whereas the second capitalizes on dealing with unknown and biased dynamics. For example, Córdova Bulens et al. (2023) show that the left arm relies more on robust control, presumably due to a less calibrated internal model of limb dynamics, whereas the dominant right arm relies much more on stochastic optimal control, which requires an accurate model of limb dynamics. OFC selects a feedback law that minimizes an expected cost, while robust control minimizes the worst‐case cost. This leads to an emphasis on system stability (robustness) over potential cost savings.

In the present study, we examined how drivers regulate their steering behavior on roads of varying widths (narrow, medium, and wide) while experiencing unpredictable, time‐varying physical perturbations. These perturbations are random and thus cannot be fully captured by an internal model. From a robust control perspective, drivers should shape their control strategies to maintain stable performance in the presence of unmodeled components of the task and plant dynamics, even when transitioning between different road widths. In contrast, if the brain can anticipate some of the perturbation structure, road width may become a primary factor of the control strategy, leading to a more efficient controller as suggested by OFC. In that case, narrower roads impose greater steering demands (Land and Horwood 1995), thus higher feedback gains, whereas wider roads permit more relaxed lower‐gain control strategies (Land and Horwood 1995; Salvucci and Gray 2004).

Here, we investigate whether, and in what manner, human control strategies guide steering across different road widths and how road width order influences the steering control policy. Our findings reveal that participants exhibited persistence in their control policy: Feedback gains were tuned primarily to favor stability rather than adjusted to changes in the cost function imposed by road width. This suggests that, under unpredictable perturbations, the human steering controller prioritizes robustness over strict optimization of the trade‐off between road‐specific task demands and cost efficiency.

## 2 Materials and Methods

### 2.1 Participants

The study was approved by the Ethics Committee of the Faculty of Social Sciences of Radboud University Nijmegen (ECSW‐2022‐082), the Netherlands. Twenty‐four naïve participants (6 male, 18 female), aged 18–41 years and with no history of motion sickness, gave written informed consent before taking part. This sample size was based on our recent study employing a similar task, which demonstrated large effect sizes with a comparable number of participants (Liu et al. 2025). Participants were compensated with course credits or €17.50. Each experimental session lasted approximately 90 min.

### 2.2 Setup

**FIGURE 1:**
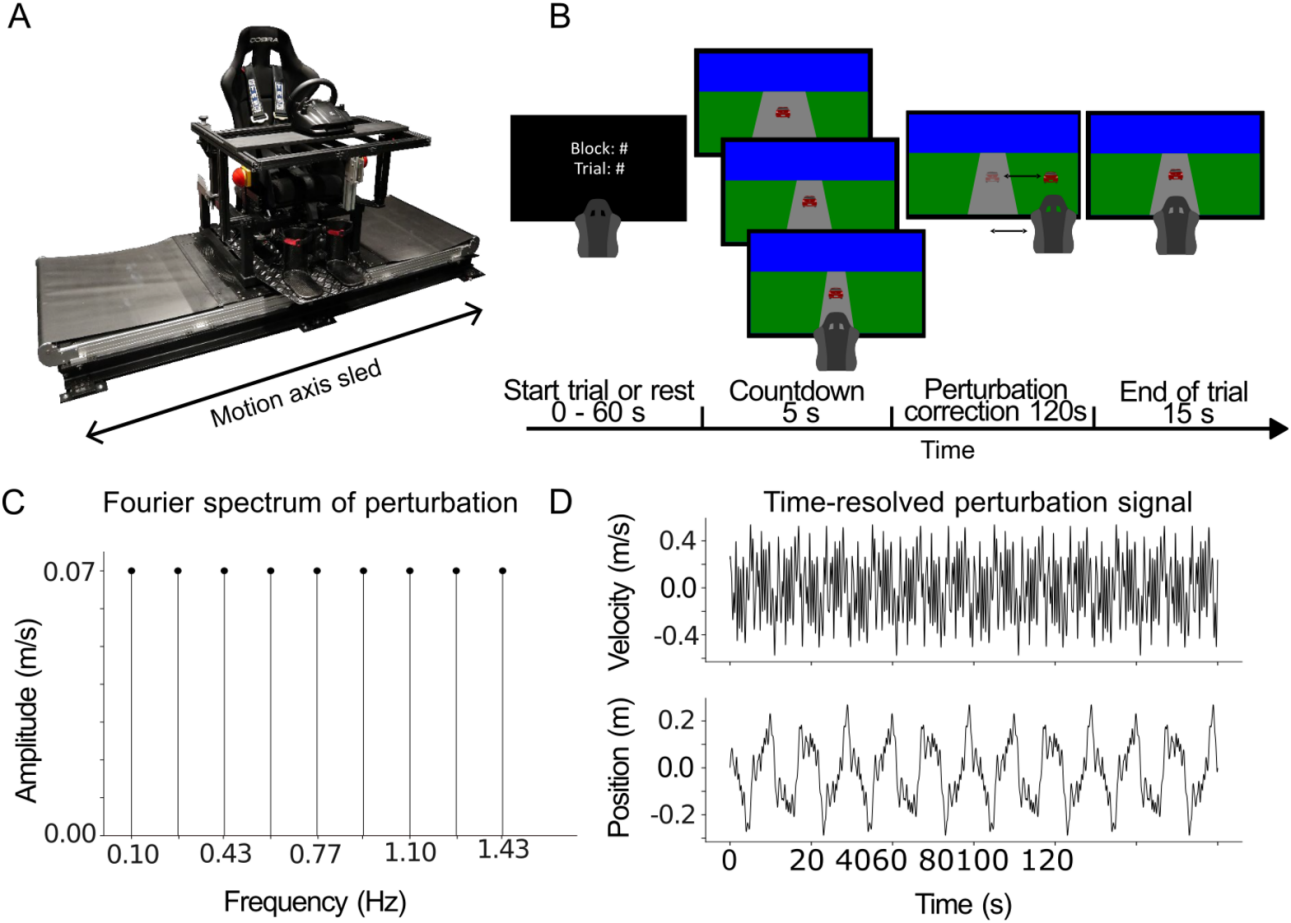
Experimental setup and paradigm. (A) The vestibular sled system, including a steering wheel. (B) Timeline of experimental procedure, including the visual display. (C) Frequency spectrum of the sum‐of‐sines perturbation. The phase of each contributing sine component was chosen pseudo‐randomly. (D) Example of time‐resolved position and velocity traces of the uncompensated perturbation signal.

Participants were seated comfortably on a custom‐built self‐steerable motion platform (dubbed the vestibular sled), where the motion axis is aligned with their interaural axis (see Figure 1A). The chair was moved along a track (0.9 m long) via a linear motor (TB15N; Tecnotion, Almelo, the Netherlands) and controlled by a servo drive (Kollmorgen S700; Danaher, Washington, DC). Emergency buttons on either side of the sled could be pressed to stop the sled motion at any time.

Participants' heads were restrained by two head clamps, and their bodies were secured by a five‐point seat belt in combination with vacuum cushions molded around the torso. Their legs and feet were strapped to reduce movement of the body within the chair. A steering wheel (Logitech G27 Racing Wheel, Lausanne, Switzerland) was mounted at a comfortable handling distance, approximately 50 cm in front of their chests. The participants placed both hands on the steering wheel at the 9 and 3 o'clock positions. The steering wheel angle, which had a range of −450° to +450° and a resolution of 0.06°, defined the instantaneous velocity of the sled (running at 100 Hz). The steering wheel angle was multiplied by 0.008 m/s/° to determine sled velocity, with the 12 o'clock direction defined as 0 m/s. Thus, a 45° rotation of the steering wheel was equivalent to a lateral sled velocity of 0.36 m/s. Sled velocity was capped at 1 m/s.

Participants faced a 77‐in. OLED screen (OLED77C3PUA; LG, Seoul, South Korea) with a resolution of 1920 × 1080 pixels and a refresh rate of 120 Hz, positioned centrally in front of the participant at 1.6 m from the cyclopean eye. The screen displayed a cartoon‐style traffic scenario at its center, featuring a road along with a vehicle (see Figure 1B). The road geometry was constructed using trapezoid polygons, each defined by four vertices. These vertices marked the boundaries of a straight road segment. The vehicle had to be steered on roads with three different widths: 0.12, 0.15, and 0.20 m, corresponding to the narrow, medium, and wide road conditions, respectively. The displayed vehicle, with its center aligned with the position of the participant on the sled track, had a width of 0.084 m (corresponding to 70%, 56%, and 42% of the respective road widths). The experiment was controlled by custom‐written Python using the PsychoPy module v.3.6.9 (Peirce 2008).

### 2.3 Behavioral Task

Participants viewed a cartoon traffic scenario, i.e., a car on a road. The lateral velocity of the sled was perturbed, which also moved the cartoon vehicle relative to the road. Participants needed to steer continuously such that the vehicle was kept within the road boundaries. No further instructions were given. They performed this task with the narrow (N), medium (M), and wide (W) roads, respectively.

The perturbation was implemented as a pseudorandom time‐varying velocity signal, constructed as a near‐zero‐mean sum of sines based on nine independent sinusoids with varying frequencies (0.1, 0.26, 0.43, 0.60, 0.76, 0.93, 1.10, 1.26, and 1.43 Hz). All frequencies had an amplitude of 0.1 m/s, and their phases were randomly drawn between 0 and 2*π*. For each trial, these phases were randomly selected with the constraint that the maximum velocity of the total perturbation did not exceed 0.6 m/s. The net platform velocity was the sum of the multisine perturbation signal and the participant's compensatory control via the steering wheel.

A trial lasted 135 s, beginning with a 5‐s countdown before the perturbation commenced. Subsequently, the velocity perturbation signal ramped up in 5 s, was at full strength for 120 s, and then ramped down over 5 s. The key segment of the trial was the 120‐s interval between the ramp‐up and ramp‐down phases, which was sufficient to quantify performance yet brief enough for the participant to stay focused on the task.

Participants were tested block‐wise on each of the three road widths, with a 5‐min break between blocks. A block started with a practice trial to familiarize participants with the task and specific road width. Thereafter, five test trials followed, with 1‐min rest between trials. Participants did not receive any feedback about their general performance at the end of a trial. Road width conditions were counterbalanced across participants, resulting in four participants tested for each of the six possible orders (NMW, NWM, MNW, MWN, WNM, and WMN).

### 2.4 Data Analysis

Data were processed offline in Python 3, using NumPy (Harris et al. 2020), SciPy (Virtanen et al. 2020), and statsmodel (Seabold and Perktold 2010) modules. We analyzed the 120‐s segments of a total of 360 trials (24 participants × 3 conditions × 5 trials). To assess overall steering performance within a trial, we computed two measures. First, we quantified task performance using the root‐mean‐square error (RMSE) of the vehicle's lateral displacement and compared it with the RMSE in the absence of any active correction (i.e., RMSE of the perturbation signal relative to the road center). Second, we assessed the percentage of time the vehicle's entire width remained within the road boundaries.

Furthermore, we analyzed the steering control dynamics through measures of (compensatory) gain and phase at the different perturbation frequencies. To this end, we used a Fourier analysis to estimate gain and phase functions (i.e., frequency response functions, FRFs) between the controlled steering velocity and the perturbation velocity signals. Gain, $G\left(f\right)$, represents the magnitude ratio of the steering velocity command and the perturbation signal, and phase, $\theta \left(f\right)$, represents the timing between the two signals as a function of frequency, f.

FRFs were computed by dividing the perturbation velocity–steering velocity cross spectrum, $P_{\mathrm{ps}}\left(f\right)$, by the perturbation velocity autospectrum, $P_{\mathrm{pp}}\left(f\right)$.

$$H\left(f\right)=\frac{P_{\mathrm{ps}}\left(f\right)}{P_{\mathrm{pp}}\left(f\right)}$$

$$G\left(f\right)=|H\left(f\right)|$$

$$\theta \left(f\right)=\tan^{-1}\left(\frac{H{\left(f\right)}_{\text{imag}}}{H{\left(f\right)}_{\text{real}}}\right)$$

Hence, as presented in a Bode plot, gain and phase are the magnitude and argument of $H\left(f\right)$, respectively. A gain of one and a phase difference of 0 indicate that the participant perfectly compensates for the perturbation, resulting in no net vehicle motion.

### 2.5 Statistics

Vehicle displacement RMSE and percentage of time on the road were each subjected to a mixed‐model ANOVA with road width magnitude (within‐subject) and road width order (between‐subject) as factors. To examine within‐trial changes, we also compared these performance measures between the first 60 s and last 60 s of the trial. The frequency‐response measures, gain and phase, showed approximately linear relationships with frequency up to about 1 Hz. To capture these relationships while respecting the appropriate constraints at zero frequency, we performed linear regressions with fixed intercepts (at one for gain and at 0 for phase). The resulting slopes from these regressions were then analyzed using mixed model ANOVAs with road width magnitude (within‐subject) and road width order (between‐subject) as factors. Unless otherwise mentioned, statistical significance was defined as *p* < 0.05. For readability, *p*‐values smaller than 0.001 are reported as *p* < 0.001; otherwise, exact values are provided.

## 3 Results

Participants steered a vehicle along roads of three different widths (narrow, medium, and wide) while being exposed to time‐varying velocity perturbations. Steering behavior was measured as the RMSE of vehicle displacement with respect to the center of the road and the percentage of time the vehicle was on the road. Steering strategy was quantified by compensatory gain and relative phase across different perturbation frequencies.

### 3.1 Overall Steering Performance

**FIGURE 2:**
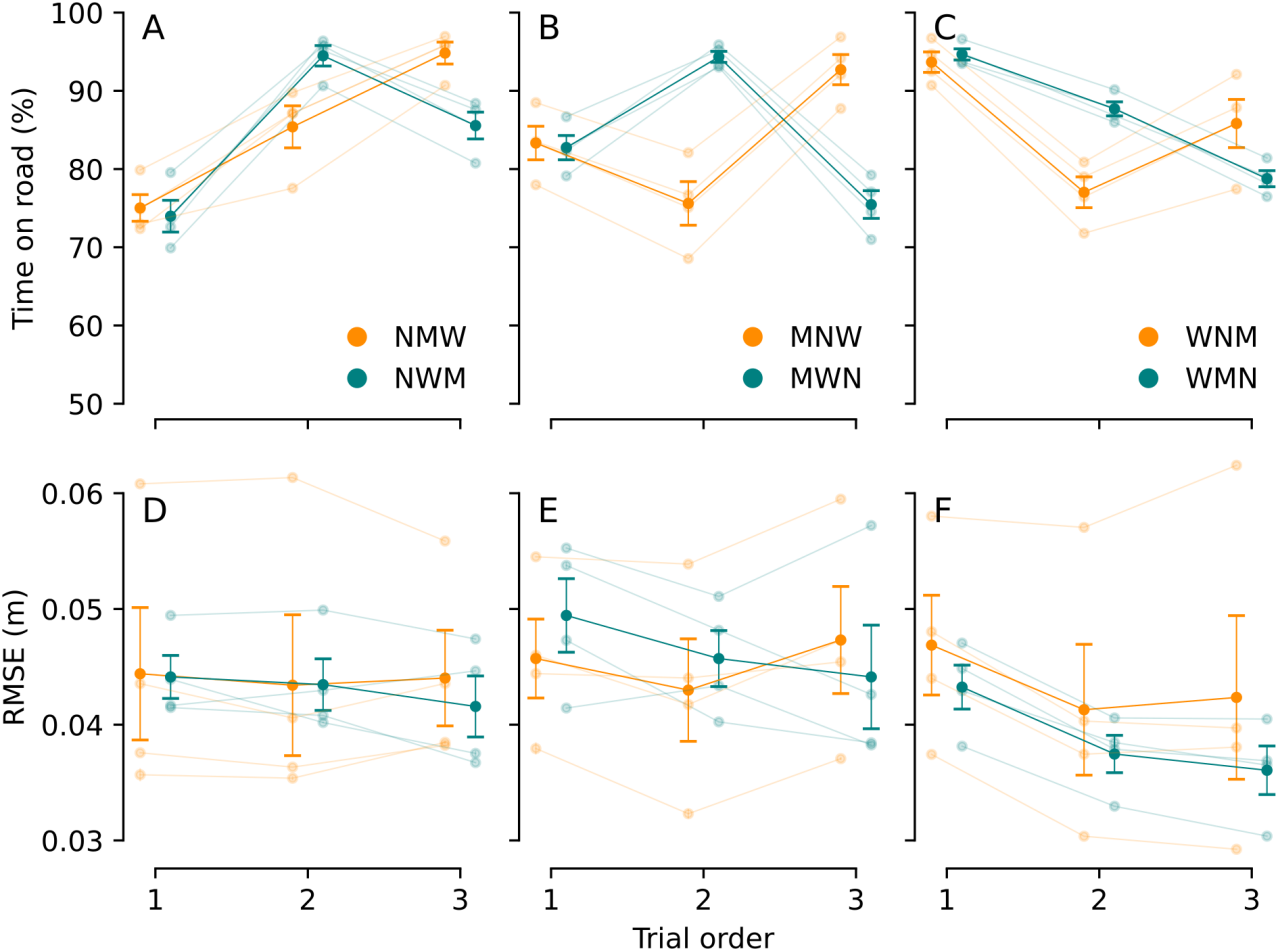
Task performance: Percentage time on road (±SE) and RMSE (±SE) for the three road widths. (A) Time on road for the two transition orders that began with the same narrow road width. (B and C) Same but transition order began with medium and wide road width, respectively. (D) RMSE for the two transition orders that began with the same narrow road width. (E and F) Same but transitions orders began with medium and wide road width, respectively. Performance of individual participants is shown in lighter colors, whereas the dark colors indicate the mean (dot) and standard error across participants. Each panel shows data for two groups of four participants.

Figure 2A–C shows the percentage of time (±SE) the vehicle remained within the road boundaries during a trial. Each of the three panels presents this measure for two transition orders that began with the same road width. Each transition order was performed by four participants. In Figure 2A, both groups started with the narrow road, for which they showed comparable performance. Next, the one group transitioned to the medium road, the other to the wide road, with the latter showing the better performance. This pattern reversed after the next transition. Figure 2B,C shows similar results for the remaining transitions. A mixed‐model ANOVA revealed a significant main effect of road width (*F*[2, 36] = 572.18, *p* < 0.001, *ηp* ^2^ = 0.97), but no significant main effect of transition order (*F*[5, 18] = 0.46, *p* = 0.79, np^2^ = 0.11) nor a significant interaction (*F*[10, 36] = 1.67, *p* = 0.12, np^2^ = 0.32). On average, participants kept the vehicle on the narrow road for 76% of the perturbation duration, for 85% on the medium‐width road, and for 94% on the wide road. This suggests that participants did not, or could not, sufficiently adjust their control strategies to obtain equal performance over road widths.

To examine the possibility of a learning effect, we compared time‐on‐road during the first 60 s of the trial with the final 60 s (i.e., 60–120 s). This analysis revealed no evidence of improvement. In fact, time‐on‐road was slightly but significantly lower in the second half of the trial (*F*[1, 23] = 17.4, *p* < 0.001, *ηp* ^2^ = 0.11).

Figure 2D–F illustrates how the RMSE (±SE) of vehicle displacement varies with road width and the order of transitions. When the narrow road is tested first (Figure 2D), RMSE remains relatively constant across road widths, suggesting robustness in control. When the wide road is tested first (Figure 2F), RMSE decreases slightly as the road narrows, indicating a tendency for participants to modulate their control strategy during these transitions. A mixed‐model ANOVA revealed a significant main effect of road width (*F*[2, 36] = 20.17, *p* < 0.001, *ηp* ^2^ = 0.53) and a significant interaction between road width and transition order (*F*[10, 36] = 4.86, *p* < 0.001, *ηp* ^2^ = 0.57) on RMSE. Like time‐on‐road, the RMSE also showed no evidence of a learning effect, as it was slightly but significantly larger in the second half of the trial (*F*[1, 23] = 29.8, *p* < 0.001, *ηp* ^2^ = 0.18). Thus, the RMSE pattern suggests that feedback gains for steering under random perturbations reflect a controller that is robust but also shows modest tuning with road width, especially when task demands become stricter.

### 3.2 Dynamics of Compensatory Control

**FIGURE 3:**
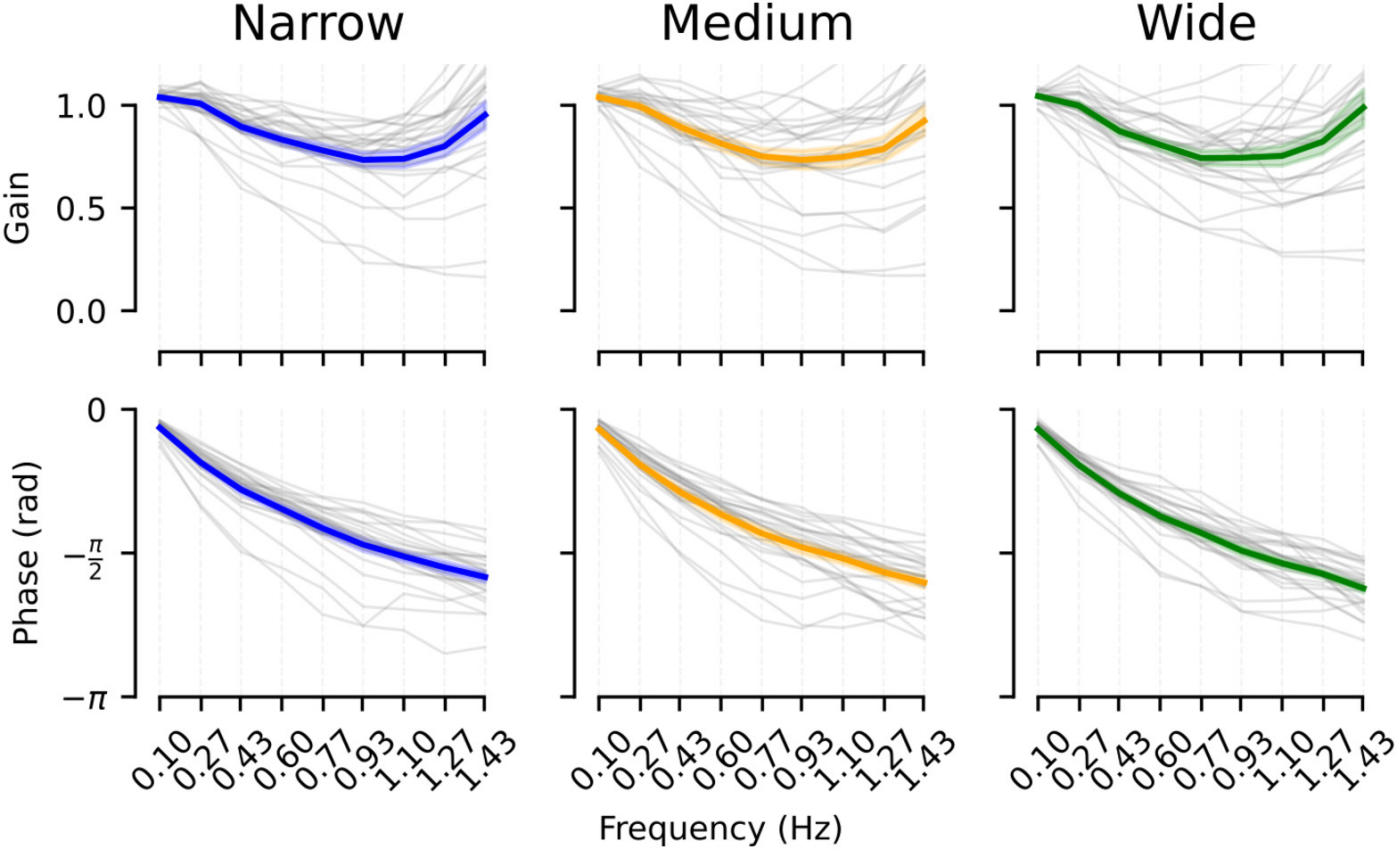
Control strategies expressed as a Bode plot. Compensatory gain (top) and relative phase (bottom) as a function of perturbation frequency, for each of the three road widths. Thin gray lines, individual participants. Colored lines: Mean across participants, with standard error as shaded band.

Although time on road and RMSE provide useful measures of steering performance, equivalent values can emerge from qualitatively different frequency response patterns, such as accurate phase with incorrect gain or vice versa. To disentangle these further, frequency‐resolved steering dynamics were quantified using compensatory gain and phase difference between the perturbation and the compensatory steering control signal (see Section 2). Perfect compensation for the perturbation is characterized by a gain of one and a phase difference of 0. Figure 3 presents the average Bode diagrams across all participants and transition orders, separately for the three road widths. The compensatory dynamics show the characteristics of a low‐pass system: a decrease in gain and a decrease in phase for frequencies up to about 1 Hz. Beyond 1 Hz, the gain starts to rise again, whereas the phase becomes increasingly less compensatory. Based on visual inspection, there appear to be no systematic effects of road width, but any of these effects may be obscured by the influence of transition order.

To statistically compare these relationships across road widths and transition orders, we summarize gain and phase as the slope of their linear relation with frequency (see Section 2). For gain, a mixed ANOVA revealed no significant main effect of road width (*F*[2, 36] = 0.32, *p* = 0.72, *ηp* ^2^ = 0.02), nor a significant interaction between road width and transition order (*F*[10, 36] = 0.71, *p* = 0.71, *ηp* ^2^ = 0.16). Because of the slight U‐shape pattern in the gain data that could obscure a possible effect, we ran an additional regression for frequencies up to 0.93 Hz, where the relationship is more closely approximated by a linear trend. Both the main effect and interaction remained nonsignificant (*F*[2, 36] = 1.59, *p* = 0.22, *ηp* ^2^ = 0.08 and *F*[10, 36] = 0.54, *p* = 0.85, *ηp* ^2^ = 0.13). For phase, where we included all frequencies, there was a significant main effect of road width (*F*[2, 36] = 3.63, *p* = 0.04, *ηp* ^2^ = 0.17), as well as a significant interaction between road width and transition order (*F*[10, 36] = 4.01, *p* < 0.001, *ηp* ^2^ = 0.53).

**FIGURE 4:**
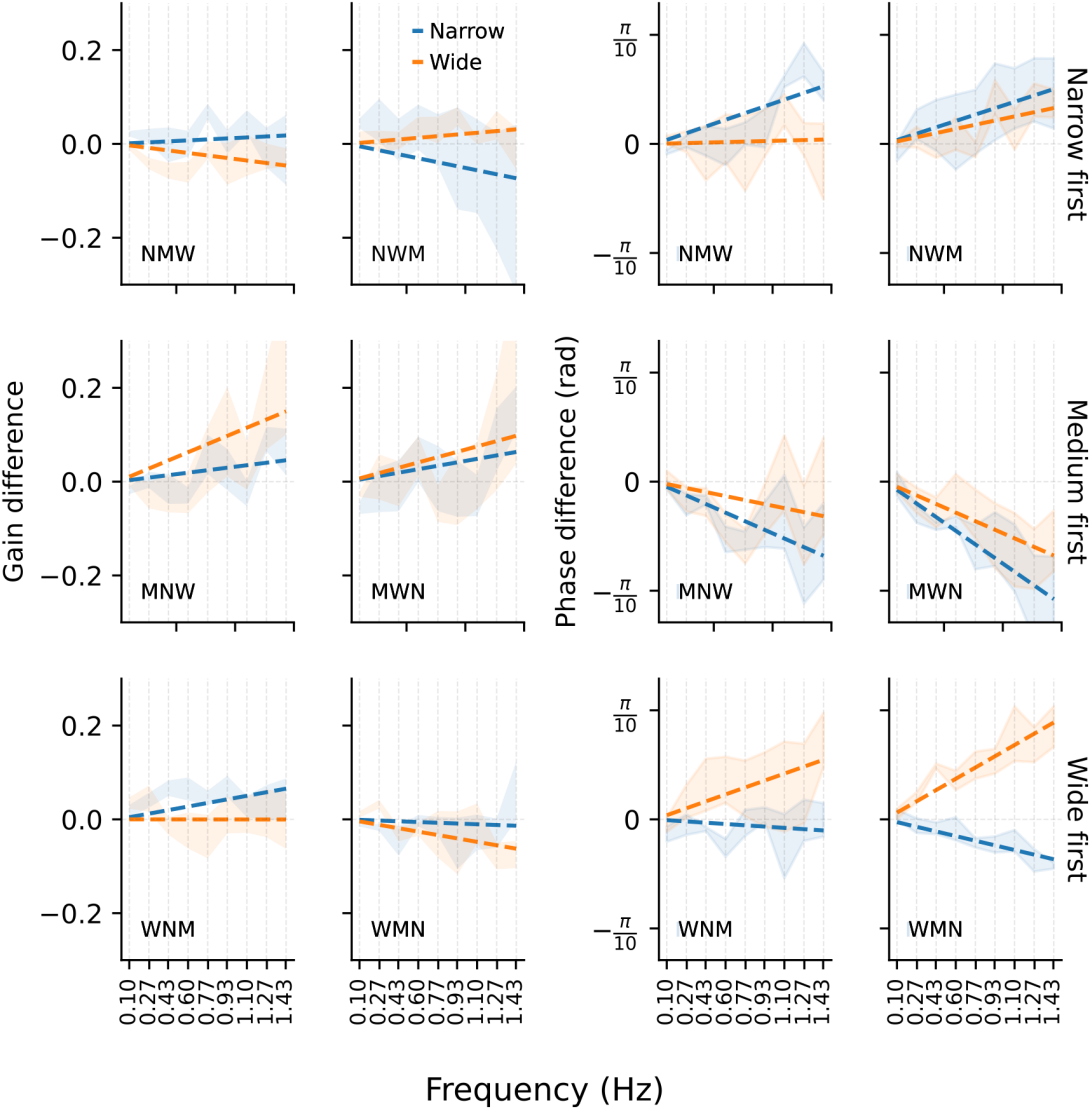
Compensatory dynamics: Gain and phase differences relative to the medium‐width road, for each transition order. Difference scores were computed for both measures between the narrow and medium roads, and between the wide and medium roads. The shaded area represents standard error.

To further examine the interaction effects between road width and transition order, we performed a further analysis for both gain and phase. For each measure, we computed the difference between the narrow and medium roads, as well as between the wide and medium roads. The resulting gain differences are shown in Figure 4A for each transition order, along with the corresponding best‐fit lines. The same computations for phase are displayed in Figure 4B. For gain, no clear pattern emerges across transition orders (the orange and blue shaded areas). In contrast, for phase, a clear separation is visible in the “Wide first” and, to a lesser degree, in the “Medium first” row. These visual patterns confirm the absence of any systematic road‐width effect on gain, while highlighting the interaction in phase. Thus, road order effects appeared primarily in phase, not gain, indicating that any road‐width adjustments in the control dynamics are expressed as shifts in control timing.

## 4 Discussion

We investigated how human participants steered a vehicle along roads of different widths (narrow, medium, and wide) with the instruction to keep it within the road boundaries despite perturbations that were presented both visually and inertially. For the small road width, results show that the vehicle crossed the road boundaries more often compared with wider roads. In terms of control, participants' steering adjustments were only slightly influenced by the sequence in which road widths were encountered. When transitioning from narrow to wide roads, participants carried over their existing strategy. This persistence was reflected in the vehicle spending more time on the wide road than on the narrow one. When transitioning from wide to narrow roads, participants largely maintained their control strategy, with only some modest modulation by road width. Regarding the frequency‐resolved compensation, road order effects were only observed in phase dynamics, suggesting that any adjustments in control policies were expressed mainly as changes in timing between perturbation and response. Overall, these results suggest that humans used a control policy that prioritizes stability (robustness) over efficiency for steering under random perturbations (Crevecoeur et al. 2019; Maurus et al. 2023).

The current random, time‐varying perturbation paradigm is well‐suited to assess the stability of the control policy. It evaluates controller robustness by injecting multiple sinusoidal disturbances with distinct frequencies and phases into the control loop. Analyzing the resulting system response allows estimation of key robustness metrics, such as gain and phase, that quantify how much variation the system can tolerate before becoming unstable. Compared with our previous study (Liu et al. 2025), the Bode diagram (Figure 3) shows a noteworthy difference in frequency‐dependent gain. In that study, we observed a systematic decrease of gain with increasing frequency. The perturbation statistics in the present work contained only the nine lower frequencies of the perturbation applied in previous work (Liu et al. 2025). This difference in the Bode diagram suggests that the control policy may also depend on the perturbation statistics (Maurus et al. 2023, 2024). In this vein, responses observed at specific frequencies should be interpreted as components of a coordinated response to the temporal and spectral structure of the perturbation. This interdependence indicates that the controller is tuned to the overall perturbation dynamics rather than reacting independently to individual frequencies.

Importantly, the present approach describes how the system reacts to sinusoidal inputs, but not where errors occur in physical space. In steering, especially on narrow roads, spatial accuracy is crucial. A controller may emphasize stability yet still permit deviations that result in crossing the road boundaries, as we showed for the narrow road (Figure 2). Thus, stability does not necessarily guarantee spatial precision: a controller can prioritize stability while failing to maintain accurate road keeping. For higher‐frequency perturbations in particular, sensory delays make corrective responses prone to overcompensation, which may worsen rather than improve performance.

The present results unveil a control architecture that emphasizes system stability (robustness) over cost efficiency. Robust control can be viewed as a model‐free design principle aimed at maximizing sensitivity, i.e., boosting feedback gains, to unpredictable disturbances (Crevecoeur et al. 2019; Maurus et al. 2023). The stability of the system is reflected in the near absence of changes in feedback gains, irrespective of which width was experienced first.

Of note, history effects in motor behaviors have been studied before in other tasks (Diedrichsen et al. 2010; Marinovic et al. 2017; Tsay et al. 2022; Verstynen and Sabes 2011; Yang et al. 2022). For example, in a stiffness stabilization task, participants utilized—in part—the stabilization strategy from the previous trial on the current trial despite the fact that they could solve the current task in an energetically more efficient way, as they would do in a blocked design (Selen et al. 2009). In bimanual reaching and stabilization tasks, the current trial strategy was also found to be influenced by the conditions of past trials (Orschiedt and Franklin 2023). In force field adaptation, where subjects were tasked to reach along a curved path, subjects were found to prefer to reach in a straight path even if more effort is required for this (Izawa et al. 2008; Kistemaker et al. 2010; Mistry et al. 2013).

In robust control, feedback strategies are expressed without relying on a detailed internal model of the expected disturbance statistics. Conversely, the perturbation statistics would need to be known a priori to compute a cost‐efficient feedback control policy, so that gains can be precisely adjusted to the road width. Indeed, OFC theory poses that gains are optimized based on a cost function that takes current task constraints into account (Todorov and Jordan 2002; Scott 2012). Although our perturbation signal was a sum‐of‐sines, it remains possible that participants anticipated certain components (e.g., the low‐frequency terms) and used this information to modestly modulate their control policy by road width. In this regard, robust control and OFC represent complementary control strategies that can coexist in the context of human steering behavior. Although robust control selects a feedback law to guarantee stability over a family of disturbances, elements of OFC modulate that law to also minimize an expected performance cost. Other work also showed that OFC and robust control are not mutually exclusive (Córdova Bulens et al. 2023). The authors demonstrated that these differing control strategies account for interlimb differences in the control and adaptation of reaching movements.

To more directly assess the potential role of cost‐efficiency in OFC in steering, one should employ a paradigm in which perturbations are more predictable, such as step perturbations (Nashed et al. 2012; Keyser et al. 2017). One suitable approach could involve participants driving a vehicle through gates of varying width, whereas occasional step‐like velocity perturbations are applied to elicit corrective steering responses. This would allow examination of how steering feedback gains incorporate the relevance of a single perturbation for steering performance.

In such a task, two key predictions derived from OFC can be tested. First, drivers are expected to allow greater natural variability in their steering when passing through wide gates, resulting in a broader distribution of passing locations during unperturbed trials. Second, in response to perturbations, drivers should exhibit reduced corrective steering, reflected as lower feedback gains when steering through wide compared to narrow gates. This follows from OFC as the principle of minimum intervention, which states that the motor system corrects only task‐relevant deviations.

Finally, in our previous work (Liu et al. 2025), we introduced a linear‐quadratic‐gaussian (LQG)–based steering model. We demonstrated that modifying the LQG control gain produced significant changes in both gain and phase of the compensatory dynamics, whereas altering the Kalman gain of the estimator affected only the phase dynamics. The present road effects were observed primarily in phase. Although phase modulation can, in principle, arise from several other sources, one possibility is that these road width effects reflect changes in the estimator, whereas the control policy itself remains unchanged. Furthermore, differences in road widths and transitions between them modulate task difficulty, attentional demands, and executive engagement. There is evidence that attention enhances the precision of sensory representations (Cohen and Maunsell 2009; Pratte et al. 2013; Engel et al. 2016). If this corresponds to a reduction in sensory noise, this would improve estimation and, in turn, change the feedback gains for steering behavior. Future studies could further dissociate the contributions from the estimator and the controller by introducing separate manipulations of sensory uncertainty and control cost.

## 5 Conclusion

We examined how human participants steered a vehicle along roads of varying width (narrow, medium, and wide) while attempting to remain within the road boundaries under visual and inertial perturbations. The findings show that steering control in the presence of such disturbances reveals a fundamental trade‐off between stability and efficiency of the control policy that depends on the imposed road‐width constraints.

## Author Contributions

**Jo‐Yu Liu:** conceptualization, data curation, formal analysis, investigation, methodology, visualization, writing – original draft, writing – review and editing. **James R. H. Cooke:** conceptualization, investigation, supervision, writing – original draft, writing – review and editing. **Luc P. J. Selen:** conceptualization, investigation, methodology, supervision, writing – original draft, writing – review and editing. **W. Pieter Medendorp:** conceptualization, funding acquisition, investigation, methodology, project administration, supervision, writing – original draft, writing – review and editing.

## Funding

This work was supported by Nederlandse Organisatie voor Wetenschappelijk Onderzoek (NWA‐ORC‐1292.19.298), Sociale en Geesteswetenschappen, NWO (406.21.GO.009), and Interreg North‐West Europe (Interreg NWE‐RE:HOME).

## Conflicts of Interest

The authors declare no conflicts of interest.

## Data Availability

All data are openly available via the Radboud Data Repository at the following link: https://doi.org/10.34973/trxj‐db04.
